# Supplementary material for: Genetic signatures of ecological diversity along an urbanization gradient
Source: PeerJ. 2016 Sep 13;4:e2444. doi: 10.7717/peerj.2444 (PMC5028742; doi:10.7717/peerj.2444)
Supplement: Supplemental Information 2 [file peerj-04-2444-s002.docx]

Supplementary material

1. Supplementary Methods Section
2. Suppl. Fig. 1 – Richness and imperviousness in data subsets.
3. Suppl. Fig. 2 – OTU richness in 1000 rarefied datasets.
4. Suppl. Fig. 3 – Principal Components Analysis on OTUs
5. Suppl. Fig. 4 – Family-level richness by imperviousness by taxonomic Class.
6. Suppl. Fig. 5 – Stacked area plot of OTU richness by taxonomic Class.
7. Suppl. Fig. 6 – Natural-history niche diversity and urbanization.
8. Suppl. Fig. 7 – Principal Components Analysis on natural-history categories
9. Suppl. Table 1 – OTU taxonomic annotations
10. Suppl. Table 2 – Natural history categorizations for Families

**Supplementary Methods**

**eDNA Collection, Extraction, and Sequencing**. We collected one-liter seawater samples from ca. 1m depth at each transect location using sampling bottles attached to a 3.3m pole. Sampling bottles were 1-L Nalgene wide-mouth bottles that had not been used previous to this study, and had been soaked in a 10% bleach solution followed by thorough rinsing with deinonized water, to minimize the risk of DNA contamination from other sources. Bottle openings were covered with 1mm mesh, also treated with bleach, to avoid capturing whole animals. Water sampling was conducted from a boat to minimize human contamination and resuspension of sediment. Water samples were kept on ice until they could be processed in the laboratory 4-8 hours later.

Our PCR protocol was as follows: a first PCR using non-indexed primers and genomic template (40 cycles); after cleaning with paramagnetic beads (Axygen BioSciences, Corning, NY, USA) the diluted (1:5) products of this reaction were used as template for a second PCR using indexed primers (20 cycles). All PCRs consisted of the following: 0.25 uL Qiagen HotStar Taq Polymerase, 2.5 uL Qiagen 10x buffer, 0.625 uL (8mM) deoxynucleotide solution, 1 uL (10 uM) each primer (forward and reverse), 18.375 uL water, and 1.25 uL template at 1:100 dilution. PCR volumes larger than 25 uL produced erratic results; therefore, we pooled uLtiple 25 uL reactions to generate PCR product for each sample sufficient for sequencing. A PCR using PCR-grade water in place of template DNA was run along with each batch of PCRs to serve as a negative control against spurious amplification. As a positive control for the PCR and sequencing protocols, we used DNA extracted from Tilapia (*Oreochromis niloticus*) tissue; this species is absent from the sampled environment. PCR thermal profiles began with an initialization step (95°C; 15 min) followed by 40 (or 20) cycles of denaturation (95°C; 15 sec), annealing (61°C; 30 sec), and extension (72°C; 30 sec).

We used the program OligoTag [1,2] to generate 25 unique DNA sequences to serve as primer indexes. These sequences consisted of 6 nucleotides each, and differed by a minimum Hamming distance of at least three. These were appended to the 5' end of both the forward and reverse primer sequences, and preceded by 3 ambiguous nucleotides (NNN). The ambiguous nucleotides not only guard against degradation of the index sequence itself, they increase diversity during initial sequencing cycles, which improves identification of clusters on the sequencing substrate (flow cell) and thus enhances the number of reads per run [3]. Thus, each indexed primer consisted of 3bp ambiguities, a unique 6bp index sequence, and a core primer sequence. The same index sequence was appended to both the forward and reverse primer sets to avoid problems associated with dual-indexed multiplexing [4]. Primers were obtained from Integrated DNA Technologies (Coralville, IA, USA).

We visualized 5 uL PCR product on a 2% agarose to confirm amplicons were absent from the negative controls, and to confirm amplicons of the correct size were present from the field samples. No negative controls produced amplicons. We quantified amplicon concentration using a QuBit fluorometer with the dsDNA HS assay (Life Technologies, Carlsbad, CA, USA).

In preparation for sequencing, we assigned amplicon samples randomly to one of 10 library index sequences, and pooled them for sequencing at equal concentrations. We prepared pooled samples (150 ng) for sequencing using the KAPA high-throughput library prep kit with real-time library amplification protocol (KAPA Biosystems, Wilmington, MA, USA), distinguishing among libraries using NEXTflex DNA barcodes (BIOO Scientific, Austin, TX, USA) following manufacturers' protocols. Sequencing was done at the Stanford Functional Genomics Facility, where 20% PhiX Control v3 was added to act as a sequencing control and to enhance sequencing depth.

**Sequence Processing and Bioinformatics**. Sequencing produced 127,608,998 paired-end reads for the 10 libraries prepared for this study. We processed these reads with a custom Unix-based script [5]. Forward and reverse reads were merged using PEAR v0.9.6 [6] and discarded if more than 0.01 of the bases were uncalled. If a read contained two consecutive base calls with quality scores less than 15 (i.e. probability of incorrect base call = 0.0316), these bases and all subsequent bases were removed from the read. Paired reads for which the probability of matching by chance alone exceeded 0.01 were not assembled and omitted from the analysis. Assembled reads were discarded if assembled sequences were not between 50 and 168 bp long, or if reads did not overlap by at least 100 bp.

Merged reads were discarded using vsearch if the sum of the per-base error probabilities was greater than 0.5 (“expected errors” USEARCH v7.0.1090) [7,8]. Sequences were demultiplexed on the basis of the 6bp index sequence at base positions 4-9 at both ends using AWK at the Unix command line. Primer sequences were removed using cutadapt v1.7.1 [9], allowing for 2 mismatches in the primer sequence. To speed up subsequent clustering, identical sequences were consolidated (dereplicated) in python. Sequences were clustered into operational taxonomic units (OTUs) using swarm v2 in fastidious mode [10], and sequences deemed to be chimeric by the default parameters in vsearch were removed (abskew 2.0, mindiffs 3, mindiv 0.8, minh 0.28) [8]. Primary quality-control and processing yielded 9.49x10^7 reads in 6197 OTU clusters.

**Contamination Removal and Sequencing-Depth Normalization**. Sequencing millions of amplicons from environmental samples invariably yields some degree of contamination, whether from laboratory, sequencing, or bioinformatic processes. In practice, there is no foolproof way of discerning rare (true) reads from low-level contamination; as a result some researchers simply set an abundance threshold below which reads are discarded [11], sacrificing some number of true OTU detections along with any putative contaminants. There is not yet a field-wide consensus on whether or how to remove potentially spurious reads from processed samples while retaining rarer OTUs, although it seems clear that post-sequencing corrections are tractable and produce reasonable results [3].

We distinguished between two primary types of putative contamination: (1) sequencing or PCR errors resulting in rare, unique reads, and (2) cross-contamination between samples of reads commonly found in the overall dataset, likely resulting from index misreads at sequencing or bioinformatic stages. There was no evidence in the dataset of a third common source of contamination, amplification of large numbers of reads from humans or other common taxa (chicken, pig, cow) that can stem from laboratory reagents or processes [12], which can dominate the underlying environmental signal in some cases.

To address the first source of putative contamination (rare, erroneous OTUs) we used a Bayesian site (or “species”) occupancy (or “occupancy detection”) modeling method, and then used both OTU identity and frequency information to address potential cross-contamination among samples.

We used a Bayesian site-occupancy modeling method to estimate the probability of the OTU representing a true positive detection, fitting a binomial distribution to OTU occurrences across replicates of each environmental sample. We eliminated from the dataset any OTU with <80% probability of being a true positive detection, leaving a total of 2846 OTUs remaining. We then detected potential cross-contamination among samples, as follows: (1) we calculated the maximum proportional representation of each OTU across all control samples, considering these as estimates of the proportional contribution of contamination to each OTU recovered from the field samples. (2) We then subtracted this proportion from each OTU in the field samples. Overall contamination was very low; for example, 99.99% of positive control reads annotated to Tilapia, with the remaining reads being small proportions (10^-5) of the same OTUs found in the field samples. The net effect of these steps was to reduce the dataset to 1812 OTUs of highest confidence, constituting 29.8×10^6 reads. The number of reads did not vary systematically between more- and less-urban sites (p = 0.25, Wilcoxon).

To standardize estimates of taxon richness across samples, we rarefied the OTUs in each sample using the smallest number of reads we observed in a single sample (124,041 reads). Here, rarefaction is statistically appropriate but conservative, in that it underestimates the total number of OTUs observed, and accordingly underestimates true differences in richness among samples. Because rarefaction is a sampling process, different rarefaction draws result in slightly different views of the ecological community. We rarefied OTU counts in each PCR replicate using rrarefy in vegan, which implements the method described in [40], generating 1000 rarefied datasets. Unless otherwise specified below, we report results from one representative rarefied dataset consisting of 11.8×10^6 reads reads representing 1664 unique OTUs; the results do not depend significantly on the choice of rarefaction replicates. For beta and gamma diversity measures, in particular, OTU identity is of importance, and accordingly we show data derived from the entire set of rarefaction replicates. Finally, for each water sample, we then averaged across the four PCR replicates to estimate the abundance of each OTU. The complete eDNA dataset is publicly available on Dryad [accession TBD upon manuscript acceptance].

Multiplexed samples from high-throughput sequencing yield different numbers of sequences per sample as a result of the limits of measurement at small volumes and of sampling during sequencing. Normalizing sequencing depth across samples is consequently necessary before comparing samples' contents. Sensitive methods of normalization—which model amplicon abundances given underlying sampling distributions—are especially important when detecting potentially small differences between large datasets, such as in RNA expression or DNA amplicon data [13], and similar methods are under development for metabarcoding studies in an ecological context [14]. Because we were interested in comparing OTU richness across samples, rather than comparing changes in abundance of a given OTU across samples, we chose to rarefy our samples as described in the main text.

96.25% of quality-controlled reads (comprising 90.9% of OTUs) could be annotated, all of which were target Metazoans. Rare OTUs were less likely to be annotatable than were common OTUs (Wilcox test, p <10^-11).

**References**

1. Coissac, E. 2012 OligoTag: a program for designing sets of tags for next-generation sequencing of multiplexed samples. In *Data Production and Analysis in Population Genomics*, pp. 13–31. Springer.

2. Boyer, F., Mercier, C., Bonin, A., Le Bras, Y., Taberlet, P. & Coissac, E. 2015 obitools: a unix-inspired software package for DNA metabarcoding. *Mol. Ecol. Resour.*

3. De Barba, M., Miquel, C., Boyer, F., Mercier, C., Rioux, D., Coissac, E. & Taberlet, P. 2014 DNA metabarcoding multiplexing and validation of data accuracy for diet assessment: application to omnivorous diet. *Mol. Ecol. Resour.* **14**, 306–323.

4. Schnell, I. B., Bohmann, K. & Gilbert, M. T. P. 2015 Tag jumps illuminated--reducing sequence-to-sample misidentifications in metabarcoding studies. *Mol. Ecol. Resour.*

5. O’Donnell, J. L. 2015 banzai. *GitHub Repos.*

6. Zhang, J., Kobert, K., Flouri, T. & Stamatakis, A. 2014 PEAR: a fast and accurate Illumina Paired-End reAd mergeR. *Bioinformatics* **30**, 614–620.

7. Edgar, R. C., Haas, B. J., Clemente, J. C., Quince, C. & Knight, R. 2011 UCHIME improves sensitivity and speed of chimera detection. *Bioinformatics* **27**, 2194–2200.

8. Rognes, T. 2015 vsearch. *GitHub Repos.*

9. Martin, M. 2011 Cutadapt removes adapter sequences from high-throughput sequencing reads. *EMBnet.journal* **17**, pp–10.

10. Mahé, F., Rognes, T., Quince, C., de Vargas, C. & Dunthorn, M. 2015 Swarm v2: highly-scalable and high-resolution amplicon clustering. *PeerJ* **3**, e1420.

11. Bokulich, N. A. & Mills, D. A. 2013 Improved internal transcribed spacer (ITS) primer selection enables quantitative, ultra-high-throughput fungal community profiling. *Appl. Environ. Microbiol.*

12. Leonard, J. A., Shanks, O., Hofreiter, M., Kreuz, E., Hodges, L., Ream, W., Wayne, R. K. & Fleischer, R. C. 2007 Animal DNA in PCR reagents plagues ancient DNA research. *J. Archaeol. Sci.* **34**, 1361–1366.

13. Love, M. I., Huber, W. & Anders, S. 2014 Moderated estimation of fold change and dispersion for RNA-seq data with DESeq2. *Genome Biol.* **15**, 550. (doi:10.1186/s13059-014-0550-8)

14. Shelton, A. O., O’Donnell, J. L., Samhouri, J. F., Lowell, N., Williams, G. D. & Kelly, R. P. 2016 A framework for inferring biological communities from environmental DNA. *Ecol. Appl.* **in press**.

Supplementary Figure 1: Mean transect-level alpha diversity (richness) for each of 1000 rarefaction draws from the overall OTU dataset, rarefied to create comparable sample sizes among sites. Variance at each site is due to stochastic variation inherent in rarefaction. Linear regression on site means, R^=0.74, p = 0.006.

Supplementary Figure 2: The OTU richness trend with imperviousness does not depend strongly on data transformation or quality-control. The same trend is evident in the normalized, quality-controlled focal rarefaction replicate (main text) as in the 100 most abundant OTUs (top) and least abundant 500 OTUs (middle) in that dataset, and in the entire, untreated raw OTU data (bottom). Regressions on site means (larger dots); original transect data shown in smaller dots.

Supplementary Figure 3. Principal Components Analysis of rarefied OTU data. Transects within sites are connected by hulls; hulls are color-coded by urbanization category (more- vs. less-urban).

Supplementary Figure 4: Number of taxonomic Families detected with eDNA for each taxonomic Class; Classes with more than 5 Families detected shown. Red trendline denotes significant quasi-Poisson (overdispersed Poisson) regression at p < 0.05; grey dashed trendline indicates nonsignificant trend. Gastropod trend is marginally significant, p=0.059. Regressions performed on site means (larger points); smaller points are transect-level data.

Supplementary Figure 5: Imperviousness and OTU richness by taxonomic Class. Classes with >20 OTUs detected are shown. Vertical light-grey lines indicate sampling locations; trends between lines are interpolated.

Supplementary Figure 6: Life-history niches and urbanization.

**Left**: The richness of life-history niches (unique combinations of habitat and natural history characteristics; defined further in the main text) in taxonomic Families detected at the eight sampled sites along a gradient of upland imperviousness. Expressed as the mean of the numbers of life histories occurring at each transect (larger circles), with transect-level data as smaller circles. Trend calculated on site means. **Right**: The same data normalized by the number of taxonomic families present at each site, yielding life histories per taxon per site. Red line indicates significant (p < 0.05) linear trend; grey line indicates nonsignificant (p = 0.1) trend. Data for focal rarefaction replicate shown.

Supplementary Figure 7: Principal Components Analysis of tri-variate life-history data for taxa identified to Family. Transects within sites are connected by hulls; hulls are color-coded by urbanization category (more- vs. less-urban).

Supplementary Table 1: eDNA OTU taxonomic assignments.

Supplementary Table 2: Natural history categorizations by taxonomic Family.
